# Challenges of Utilizing Medical Big Data in Reproductive Health Research

**DOI:** 10.3389/frph.2022.800760

**Published:** 2022-03-16

**Authors:** Tianyu Dong, Min Zhu, Rui Li, Xu Wang

**Affiliations:** ^1^Tripod (Nanjing) Clinical Research Co., Ltd., Nanjing, China; ^2^Department of Health IT Solution, Shanghai Synyi Medical Technology Co., Ltd., Shanghai, China; ^3^Department of Endocrinology, Children's Hospital of Nanjing Medical University, Nanjing, China

**Keywords:** reproductive health, clinical research, big data, artificial intelligence, children, women

## Abstract

In the background of the “Three-Child Policy” introduced by the Chinese government, reproductive health has become one of the most important public health issues. With the promotion of digitization management of medical care institutions for women and children in the country, there will be chances to acquire medical big data of obstetrics and pediatrics. Here the authors are presenting their opinions on the challenges of the management and utilization of reproductive big data.

## Introduction

With the further understanding of Developmental Origins of Health and Disease (DOHaD) theory, early-life is considered as a key period of the whole life health management. Substantial research data have demonstrated the association between early-life and long-time health (1), which emphasizes the particular importance of reproductive health. In the background of the “Three-Child Policy” introduced by the Chinese government, reproductive health has become one of the most important public health issues.

The mode of medical service has changed with the introduction of the policy of accelerating digital development in China. The concept of “Medical Big Data” has come into the scope of the public due to the promising values for improving the quality of medical care and conducting clinical research (2). Health-care professionals, clinical researchers and even the politicians are thinking of how these data should be acquired, managed and used. With the digital development and transformation of the Information System in medical institutions for women and children, medical data of reproductive health need to be fully understood and handled in the context of “Medical Big Data” (3).

## Challenges of the Data Management

Usually, clinical information from the medical institutions for women and children's health include medical records, medical orders, nursing, testing, imaging, and pathology, etc. From the perspective of the data manager, the raw information from different functional units should be extracted, cleaned, linked and stored in the cloud of a single data management platform to generate standardized and structured datasets. As the volume, velocity, veracity and variety characters of increasing medical data, the medical engineering and artificial intelligence (AI) techniques are required in large-scale studies (4). There are several challenges during the medical data governance process: data integrity and extraction stage need to improve the accuracy and data precision during; disease data sets define and normalization can improve medical data efficiency; Natural Language Processing (NLP) technologies help to access and abstract from unstructured data; the machine-learning algorithms to reach to those converted structure data to detect diseases and perform more precision studies (5). NLP is the mainly technology to abstract precision terms from narrative clinical reports and medical big data. The “concepts” need to be defined to recognize the terms. According to different study objectives, the manuscript can be used to the appropriate research field (6). The named entity recognizer (NER) can identify those concepts in clinical free-text notes. Then a relation extraction (RE) method can identify the relations between them. For NER, there has several deep learning-based approaches with embedding various to influence the different results (7). The methods and evaluation will be conducted with mimic data.

Another prominent problem for medical data could be the solution to ensuring the data consistency, integrity and accuracy, as the medical records from a single individual are usually collected from multiple sources especially for children. Unified coding is a prerequisite for matching information from different medical institutions (e.g., records from women's health institutions to children's hospitals) and/or functional units within an institution. Parents' IDs are used as the only identification for babies as they usually do not have their names or IDs at birth. They are also used to track the family history and should be linked with future children's IDs. Identifying information should be uniquely coded, and effective unified coding helps with integration of information according to the flow direction and logical relationship when dealing with data format, repetition, attribute value error, inconsistency and other problems. Confidentiality of private information is of great importance in the modern age (8). Medical records should be anonymized to protect the privacy and information security of patients. On the premise of strict hardware guarantee, the maintenance system and operation standard need to be formulated. The database access authority, administrator authority (7) and docking user authority should be set. Identification information is only assessable to qualified staff and should be stored separately with medical data. It is necessary to monitor the operation of users in order to effectively prevent the human errors leading to unexpected information disclosure.

## Utilization of Medical Big Data

The utilization of Medical Big Data is still in its early development period in China. It is recommended that clinical researchers cooperate with clinicians to generate good scientific research questions. Clinicians are familiar with the problems in the area of reproductive health that need to be solved, and clinical researchers, including epidemiologists and biological statisticians, can provide valuable suggestions on the feasibility and performance of the study. The database should be user friendly for searching for information and summarizing subsets of the whole dataset. Reproductive health data are collected from parents-child pairs, which makes the datasets very complex and hard to explore the causality. Traditional statistical methods such as logistic regression and survival analysis are still very useful, but sometimes cannot meet the needs of mining complex data. The values of machine learning in data mining have been demonstrated in plenty of previous epidemiological studies (9–11). Novel tools are warranted to model the multivariate information. Taking the advantages of machine learning and big data, researchers are able to obtain robust results and simulated extrapolation in more common populations.

## Discussion

Reproductive health is always a concern in modern societies due to the aging population. Great attention is paid to women and children's health in China. Several large birth cohorts have been constructed in the country in recent years. However, the utilization of unstructured medical data are still under development. With the promotion of digitization management of medical care institutions for women and children, there will be chances to acquire medical big data of reproductive health. These data could be well used for researches in the context of good data standardization. AI techniques will help with both the data structuring and data analysis/interpretation. The accurate identification of babes and their parents is an important issue of ensuring the data consistency and integrity. Confidentiality of private information should be protected seriously especially for children. In addition to the collection of medical records, biobanks are promising for further development in the area. Biobanks for samples collected from parent-child pairs enables the employment of advanced systems biology techniques and special study design for discovery of intergeneration effects at the molecular levels. These precious resources will contribute to future reproductive researches, and greatly promote the health care of women and children.

## Data Availability Statement

The original contributions presented in the study are included in the article/supplementary material, further inquiries can be directed to the corresponding author/s.

## Author Contributions

XW designed the work and made critical revisions on the manuscript. TD drafted the manuscript. MZ and RL made critical revisions on the manuscript. All authors contributed to the article and approved the submitted version.

## Conflict of Interest

TD was employed by Tripod (Nanjing) Clinical Research Co., Ltd. MZ and RL were employed by Shanghai Synyi Medical Technology Co., Ltd. The remaining author declares that the research was conducted in the absence of any commercial or financial relationships that could be construed as a potential conflict of interest.

## Publisher's Note

All claims expressed in this article are solely those of the authors and do not necessarily represent those of their affiliated organizations, or those of the publisher, the editors and the reviewers. Any product that may be evaluated in this article, or claim that may be made by its manufacturer, is not guaranteed or endorsed by the publisher.
